# Functional characterization of castor bean (*Ricinus communis*) DGAT3 and DAcT enzymes in *Arabidopsis thaliana*

**DOI:** 10.1186/1753-6561-8-S4-P117

**Published:** 2014-10-01

**Authors:** Thomaz Trenz, Andréia Turchetto-Zolet, Márcia Margis, Rogério Margis, Felipe Maraschin

**Affiliations:** 1 Universidade Federal do Rio Grande do Sul, Sapiranga, Brazil

## Background

Triacylglycerols (TAGs) are the main seed storage lipids of plants. TAGs chemichal properties are largely dependent on their fatty acid composition. Diacylglycerol Acyltransferase (*DGAT*) genes encode the main enzymes needed for TAG biosynthesis. Different types of *DGAT *genes (named *DGAT*1, *DGAT*2, *DGAT*3 and *DAcT*) have been identified in plants. *DGAT*1 and *DGAT*2 are well characterized, but little is known about *DGAT*3 and *DAcT *genes in most plant species. The understanding of TAG biosynthesis enzymatic steps and its transcriptional regulation in plants is important to help improve the content and composition of nutritional and industrial oils. DAcT or Diacylglycerol-acetyltransferase was previously identified in *Euonymus alatus *and it seems to be related to DGAT enzymes. This enzyme catalyzes the condensation of acetil-CoA to diacylglycerol for the formation of 1,2-diacyl-3-acetyl-sn-glycerols or simply "ac-TAGs". These sn-3 acetylated diacylglycerol oils are abundant in *Euonymus *and display a 30% reduction on viscosity. DGAT3 enzymes were first reported in peanut and are unique due to their cytoplasmic localization due to the lack of transmembrane domains.

## Methods

Our goal is to identify and characterize DGAT3 and DAcT genes of castor bean (*Ricinus communis*). The cDNA sequences from *Euonymus alatus *and *Arachis hypogaea *were used as queries in the blastx and tblastx programs to search for DAcT (Diacylglycerol acetyltransferase) and DGAT3 (soluble Diacylglycerol acyltransferase) from different plant species. The sequences identified as RcDAcT and RcDGAT3 in the *Ricinus communis *genome were used to design RT-qPCR primers to evaluate their expression profile in developing seeds. For the functional characterization of these proteins, full cDNA sequences were amplified with gene-specific primers and cloned in pENTR-D. These entry clones were C-terminally fused to the YFP and CFP coding sequences. *Arabidopsis *mesophyll protoplasts were transformed with the YFP construction to identify DGAT3 and DAcT subcellular localization. Via Floral-dip, we transformed castor bean DGAT3-CFP in *Arabidopsis *to characterize its role in lipid metabolism and to obtain a purified protein for enzymatic assays. We also transformed yeast lipid synthesis mutants to confirm the role of these proteins in TAG accumulation by complementation assay.

## Results and conclusions

We have successfully identified 4 different DAcT paralogs and one DGAT3 ortholog in castor bean. We have already characterized the expression pattern of the DGAT3 gene in castor bean in five different stages of castor bean seed development where we verified an active expression of this gene; however, by RT-qPCR approach, it was not possible to identify substantial expression for DAcT genes in castor bean developing seeds. The DAcTA-YFP fusion protein seems to be localized to the ER membrane as the other previously described DGATs are, on the other hand, DGAT3-YFP seems to localize to cytoplasmic structures which we believe to be oil bodies. Currently, we are characterizing *Arabidopsis *transgenic lines overexpressing castor bean DGAT3-CFP and evaluating the capacity of DGAT3 and DAcT genes to complement yeast lipid-synthesis mutants.

